# Immunomudulatory effects of hydroalcoholic extract of *Hypericum perforatum*

**Published:** 2015

**Authors:** Seyyed Meysam Abtahi Froushani, Hadi Esmaili gouvarchin Galee, Mahsa Khamisabadi, Bita Lotfallahzade

**Affiliations:** 1 *Assistant Professor of Immunology, Department of Microbiology, Veterinary Faculty, Urmia University, Urmia, Iran.*; 2 *MSC Student of Immunology, Department of Microbiology, Veterinary Faculty, Urmia University, Urmia, Iran.*; 3 *Student of Veterinary Medicine, Veterinary Faculty, Urmia University, Urmia, Iran.*

**Keywords:** *Hypericumperforatum*, *Humoralimmunity*, *Cellularimmunity*, *Lymphocyteresponse*

## Abstract

**Objective:** *Hypericum perforatum* (St. John's Wort) has long been used in traditional medicine to treat a variety of internal and external ailments. The present study was done to evaluate the immumodulatory potentials of the hydroalcoholic extract of *H. perforatum*.

**Materials and Methods:** Twenty male BALB/c-mice were randomly allocated in two equal groups and immunized with sheep red blood cells (SRBCs) and complete Freund’s adjuvant. Mice in the treatment group orally received hydroalcoholic extract of *H. perforatum* (110 mg/Kg daily) from the beginning of the study which continued for 2 weeks.

**Results:** The data indicated a significant increase in the level of anti-SRBC antibody and simultaneously a significant decrease in the level of cellular immunity, an enhancement in foot pad thickness, in treatment group compared to control group. The level of the respiratory burst in phagocytic cells and the level of lymphocyte proliferation in splenocytes were significantly decreased in the treatment group compared to control group. Moreover, extract caused a significant reduction in the production of pro-inflammatory IL-17 as well as IFN-γ, parallel to increasing the level of IL-6.

**Conclusions:** The hydroalcoholic extract of* H. perforatum* may be used as a natural source for treatment of immunopathologic conditions.

## Introduction

*Hypericum perforatum* (St. John's Wort) is a perennial flowering plant and has been used for centuries as a natural remedy for the treatment of a variety of internal and external ailments (Birt DF et al., 2009 ▶; Huang N et al., 2013 ▶)*. H. perforatum* contains numerous compounds with biological activity such as hypericin, pseudohypericin, flavonoids, oligomeric procyanidines and hyperforin (Wentworth JM et al., 2000 ▶; Nathan PJ, 2001 ▶). Extract from H. perforatum has been used as a topical remedy for treatment of wounds, abrasions, burns, and muscle pain (Reuter J et al., 2008 ▶). Hyperforin, a major constituent chemical of *H. perforatum* has been shown to have antibacterial properties against gram-positive bacteria (Cecchini C et al., 2007 ▶) and also may be useful for treatment of alcoholism (Kumar V et al., 2006 ▶; Reuter J, et al., 2008 ▶). Hypericin and pseudohypericin have shown both antiviral and antibacterial activities (Huang N, et al., 2013 ▶). It has been demonstrated that hydroalcoholic extract of *H. perforatum* could be beneficial in the management of hyperlipidemia and atherosclerosis (Asgary S et al., 2012 ▶). However, the original use of H*. perforatum *in traditional medicine is treatment of patients with depression disorders (Wentworth P, Jr. et al., 2003 ▶; Dwyer AV et al., 2011 ▶; Asgary S, et al., 2012 ▶). Hypericum has been already a popular antidepressant drug in many countries (Dwyer AV, et al., 2011 ▶).

Immunomodulation is desired when the host defense mechanism has to be activated under the immunodeficiency situations or when a selective immunosuppression is required in conditions such as autoimmune disorders (Visavadiya NP et al., 2009 ▶; Mitra Mazumder P et al., 2012 ▶). Nowadays, medicinal plants with immunomodulatory capacities offer new horizon in traditional medicine (Visavadiya NP, et al., 2009 ▶). Accordingly, the present study was conducted to investigate the immumodulatory potentials of the hydroalcoholic extract of *H. perforatum*.

## Materials and Methods

**Materials**

RPMI 1640 and fetal calf serum were bought from GIBCO/Life Technologies Inc. (Gaithersburg, MD). Nitro blue tetrazolium, dioxin, complete Freund’s adjuvant (CFA), dimethyl sulfoxide (DMSO), phytohemagglutinin (PHA), and 3-[4,5-dimethylthiazol-2-yl]-2,5-diphenyl tetrazolium bromide (MTT) were obtained from Sigma-Aldrich (St. Louis, MO). RPMI 1640 and fetal calf serum were bought from GIBCO/Life Technologies Inc. (Gaithersburg, MD). The cytokine assay by enzyme-linked immunosorbent assay (ELISA) kits for interferon gamma (IFN-γ), interleukin (IL)-6, and IL-17 were procured from Bender MedSystems (Vienna, Austria). 

**Animals**

The male inbred BALB/c mice (5–6 weeks old, 20–25 g) were purchased from the Pasteur Institute of Iran. Animal welfare was observed in compliance with the regulations of the Iranian Ministry of Healthcare approved by the Medical Ethics Committee for Animal Studies. The animals were housed in an automatically controlled room under conditions of optimized light (12:12 light-dark cycle), humidity (55-60%), and temperature (22-23 °C). Under the optimized conditions, the animals were maintained on a standard diet. The acclimatization period at the above conditions was established, at least, for one week.

**Extraction of** ***H. perforatum***

Six grams of dried and ground *H. perforatum* (Collected from Yasuj, Iran) were extracted for 6 h through Soxhlet with 500 ml 95% ethanol. The extract was then filtered and subsequently dried by rotary evaporation at 40 °C followed by lyophilization. The dried extract was dissolved in distilled water and propylene glycol (4:1) and stored without light exposure at −20 °C.

**Experimental design, Immunologicalimmunological challenge, and evaluation**

Mice were randomly allocated into 2 groups: control mice and treatment group. Each group had 10 animals. Since the experiment began, animals were intraperitoneally immunized twice with one week interval by 1×10^9^ sheep red blood cells (SRBC) emulsified in CFA. Mice were bled from their hearts 5 days after the last injection and the levels of anti-SRBC antibody were measured by the microhemagglutination test as described previously (Mitra Mazumder P, et al., 2012 ▶).

 Moreover, 48 h before bleeding time, 1×10^9^ SRBCs in 50 μl of PBS were administered subcutaneously into the left hind foot pad of each mouse and simultaneously the same volume of PBS was injected into the right foot pad as a negative control. 

Footpad thickness was measured before bleeding time with a dial caliper and the mean percentage increase in footpad thickness was measured according to the following formula: [(Thickness of left footpad) - (Thickness of right footpad) × 100] / (Thickness of right footpad).

Hydroalcoholic extract of *H. perforatum* (110 mg/Kg daily) was intraperitoneally injected into the treatment group from the beginning of the study (onset of immunization) and continued throughout the study when the mice were bled. Control mice received an equal volume of distilled water containing propylene glycol with the similar schedule as treatment group.

**Cytokines production**

Spleen cells were aseptically isolated from mice at bleeding time. In brief, single-cell suspensions of splenocytes were prepared in RPMI 1640 medium supplemented with 10% fetal calf serum and red blood cells (RBCs) were removed by RBC lysis buffer. Next, cell suspensions (2×10^6^ cells/ml) were incubated in 24-well plates and pulsed with 50 μL PHA solution (1 mg/ml). The culture supernatants were collected after 72 h. IFN-γ, IL-17, and IL-10 production were assumed by ELISA according to the manufacturer's instructions 

**Splenocytes proliferation**

Proliferation potential of lymphocytes in splenocyte population was evaluated by MTT assay. The splenocytes were plated in 96-well flat-bottomed plates in RPMI 1640 medium supplemented with 10% fetal calf serum (1×10^5^ cells/100μl/well) and stimulated with 50 μL PHA solution (1 mg/ml) or medium alone. After 72 h incubation, cultures were pulsed with 20 μl of the MTT solution (5 mg/ml) for 4 h at 37 °C. Then, 150 ml DMSO was added and shaken vigorously to dissolve formazan crystal. The optical density (OD) at 550 nm was measured using microplate reader (Dynatech, Denkendorf, Germany). The experiments were done in triplicate sets. The results were expressed as the proliferation index according to the ratio of OD_550_ of the stimulated cells with PHA to OD_550_ of the non-stimulated cells.

**Respiratory burst in splenocyte population**

Respiratory burst of phagocytic cells in splenocyte population was checked using NBT dye reduction as described previously with some modification (Müller J et al., 1981 ▶; Nabi AH et al., 2005 ▶; Hamaliaka A and Novikova I, 2010 ▶). In brief, 100 μl of suspension of splenocytes with 0.1 ml of *S. aureus* suspension (10^8^ cell/ml) and 0.1 ml of 0.1% NBT in PBS (pH = 7.4) were mixed. The mixture was incubated at room temperature for 15 min and subsequently kept at 37 °C for additional 15 min. The reduced dye was extracted in dioxan and quantitated at 520 nm.

**Statistical analysis**

Data were analyzed using Student's t-test and presented as means ± SD. P-values less than 0.05 were considered statistically significant.

## Results

Hydroalcoholic extract of Hypericum perforatum diminished cellular immunity and concurrently potentiated humoral immunity.

Footpad thickness after challenge with SRBC was performed as an indicator for evaluation of delayed type of hypersensitivity (DTH) reaction. As shown in Table 1, hypericum-treated mice showed significantly lower DTH responses than the control mice. Conversely, mean antibody titer in the treatment group was significantly higher than the mean antibody titer in control mice (Table 1).

**Table 1:** Effects of hydroalcoholic extract of *Hypericum perforatumon* on humoral and cellular (Percentage of footpad thickness) immunity.

**Group**	**Antibody titer**	**Percentage of footpad thickness**
Treatment	209 ± 12.44	19.06 ± 2.32
Control	53.19 ± 5.11	46.2 ± 3.02
P-value	< 0.001	< 0.01

**Figure 1:**
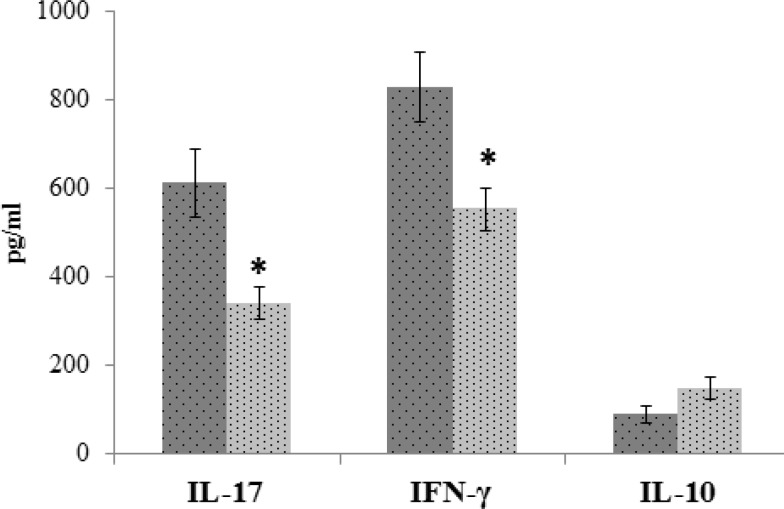
Cytokines production assay after treatment with hydroalcoholic extract of *Hypericum perforatum*. Spleen cells isolated from immunized mice with SRBC were cultured with 50 µl of PHA (1 mg/ml) for 72 h. The levels of IFN-γ, IL-17, and IL-10 in culture supernatants were determined after 72 h by ELISA. The results were shown as mean±SD. (* p < 0.001, versus control mice).

Administration of extract alleviated production of IL-17, IFN-γ, lymphocyte proliferation index, and NBT dye reduction test. A significant decrease in secretion of IL-17 and IFN-γ in cells from extract-treated mice were found compared to cells from vehicle-treated group (Figure 1). The level of IL-10 diminished in the treatment group but this reduction was not significant (Figure 1).

However, the proportions of INF-γ to Il-10 or IL-17 to IL-10 were decreased significantly (Table 2).

**Figure 2:**
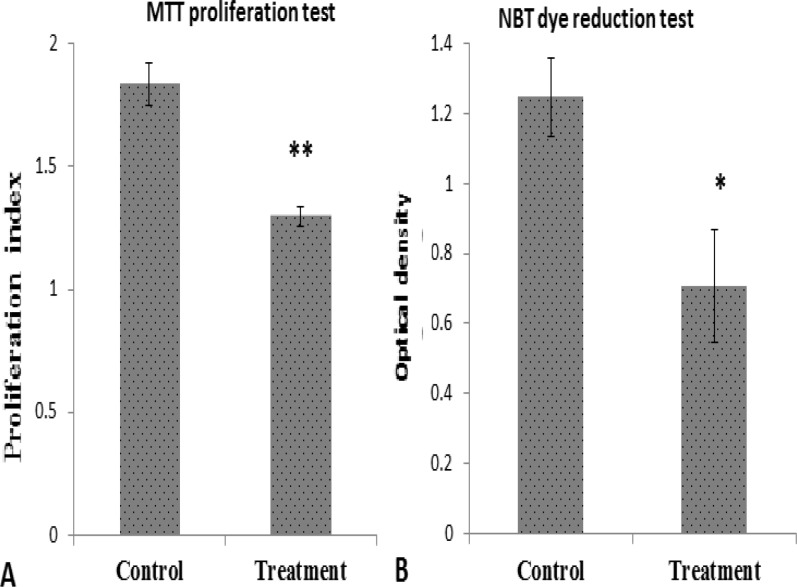
Effects of hydroalcoholic extract of *Hypericum perforatum* administration on lymphocytes proliferation and respiratory burst in phagocytic cells. Splenocytes were isolated from sensitized mice with SRBC**. **A) Splenocytes cultured with 50 μL PHA solution (1 mg/ml) for 72 h**. **Then, lymphocytes proliferation were evaluated by MTT test. B) Splenocytes with *S. aureus *suspension and NBT were mixed and incubated for 30 min as detailed under materials and methods. The reduced dye was extracted in dioxan and quantitated at 520 nm. The values were presented as mean ± SD. (*p<0.01, ** *p*<0.001 versus control mice).

Moreover, a significant reduction in splenocyte proliferation and respiratory burst was observed in extract-treated mice compared to the normal control animals (Figure 2).

**Table 2:** Cytokine ratio between IFN-γ: IL-10 and/or IL-17: IL-10.

**Cytokines ratio Group**	**IFN-γ:IL-10**	**IL-17:IL-10**
Treatment	6.16 ± 0.8	4.716± 0.94
Control	21 ± 3.22	14.34± 1.8
P -value	< 0.001	< 0.001

Splenocytes separated from immunized mice with SRBC were incubated with 50 µl of PHA (1 mg/ml) for 72 h. The ratio of IFN-γ to IL-10 and the ratio of IL-17 to IL-10 in culture supernatants were determined after 72 h. The data were shown as mean±SD.

## Discussion

*H. perforatum* has been found to have anti-inflammatory properties due to its inhibitory benefits on the expression of pro-inflammatory genes like cyclooxygenase-2 and inducible nitric-oxide synthase (Kraus B et al., 2010 ▶; Huang N et al., 2012 ▶).Obviously, inflammatory response and immune function are completely intertwined. DTH is one of the typical response patterns of T cell-mediated immunity and causes T cell–dependent inflammation (Kobayashi K et al., 2001 ▶). The first requirement for DTH reaction is the priming of a special effector class of antigen specific T cells (Kuerten S and Lehmann PV, 2011 ▶). DTH response has long been believed to be mediated by Th1 cells (El-behi M et al., 2010 ▶; Murdaca G et al., 2011 ▶). However, this concept was doubted because mice with deficiency deficient in components of the IL-12/Th1 axis including IL-12α (IL-12p35), IFN-γ, or IFN-γ receptor were more susceptible to autoimmunity. This deficiency in understanding immunopathology was resolved by the discovery of IL-17-producing CD4^+^ T cells (Th17) (Aranami T and Yamamura T, 2008 ▶; El-behi M, et al., 2010 ▶; Fletcher JM et al., 2010 ▶). IL-17 (also called IL-17A) has a potent pro-inflammatory property (Korn T et al., 2007 ▶) and is a crucial factor for the promotion of DTH induction (Kuerten S and Lehmann PV, 2011 ▶). It seems that Th17 cells initiate the inflammatory response, while Th1 cells determine the tissue damage (Murdaca G, et al., 2011 ▶). After immunizations with CFA, killed mycrobacteria produce the microenvironment for the Th17 and Th1 polarization of the T cells that are specific for the antigen mixed into the adjuvant (Kuerten S and Lehmann PV, 2011 ▶). It has been demonstrated that IL-6 participates in Th17 cell polarization (Dong C, 2009 ▶).

Our results showed that treatment with hydroalcoholic extract of *H. perforatum* after challenge with SRBC significantly suppressed potent pro-inflammatory cytokines (IL-17, IFN-γ, and IL-6) and consequently DTH reaction. The level of IL-10, a cytokine with anti-inflammatory properties, did not show any significant differences between groups. However, the proportions of INF-γ to Il-10 or IL-17 to IL-10 were changed in favor of IL-10.

These findings offer new insight into the potential mechanisms underlying the immumodulatory effects of hydroalcoholic extract of *H. perforatum*. Monocytes/macrophages are the second players in DTH reaction (Kobayashi K, et al., 2001 ▶). Significant reduction in IL-17 and IFN-γ production could somewhat explain the decrease of the respiratory burst in mononuclear phagocytic cells in mice treated with *H. perforatum*. Pervious data also showed that *H. perforatum* inhibited LPS-induced production of inflammatory mediators, including prostaglandin E2 and nitric oxide (NO) in activated macrophages (Huang N, et al., 2012 ▶).

In general, cellular and humoral arms of immunity are reciprocally regulated (Kobayashi K, et al., 2001 ▶). Therefore, increasing the humoral immune response following reducing DTH reaction in the *H. perforatum* treatment group is not far-fetched. Interestingly, a recent document demonstrated that hydroalcoholic extract of *Hypericum perforatum* administered as a dietary supplement in the peri-immunization period potentiated the humoral response of hens to the influenza vaccine (Jiang w et al., 2013 ▶). Altogether, immune deviation from cellular immunity to humoral responses was concurrent with a significant decrease in lymphocyte proliferation. T cell-mediated immunity has a substantial role in determining the extent of organ-specific autoimmune diseases (Aranami T and Yamamura T, 2008; ▶ Kuerten S and Lehmann PV, 2011 ▶). Therefore, these results suggest that extracts from *H. perforatum* may be a promising strategy to treat organ-specific autoimmune diseases.

The interaction between immune and nervous systems plays a substantial role in the pathophysiology of depression (Berk M et al., 2013 ▶; Munzer A et al., 2013 ▶). Depression disorders are related to a chronic, low-grade inflammatory response, and activation of cell-mediated immunity (Berk M, et al., 2013 ▶). Patients with depression show increased production of pro-inflammatory cytokines such as IL-6 (Kim JW et al., 2013 ▶; Munzer A, et al., 2013 ▶). On the other hand, *H. perforatum* is widely used for treatment of depression (Wentworth P, Jr., et al., 2003 ▶; Dwyer AV, et al., 2011 ▶; Asgary S, et al., 2012 ▶). Interestingly, our results showed that the hydroalcoholic extract of *H. perforatum* significantly diminishes cellular immunity and production of pro-inflammatory cytokines.

The in vivo immunomudlatoty effects of *H. perforatum *may be partly due to inhibition of pro-inflammatory cytokine IL-17, IFN-γ, and IL-6. However, other mechanisms may also be involved and these remain to be clarified. Finally, this data suggest that the hydroalcoholic extract of *H. perforatum* may be used as a natural source to intervene in immune system.
